# Ecological Validation and Reliability of Hexoskin Wearable Body Metrics Tool in Measuring Pre-exercise and Peak Heart Rate During Shuttle Run Test in Professional Handball Players

**DOI:** 10.3389/fphys.2020.00957

**Published:** 2020-07-31

**Authors:** Monoem Haddad, Souhail Hermassi, Zlatan Aganovic, Feriel Dalansi, Mariam Kharbach, Azam Omar Mohamed, Khalid W. Bibi

**Affiliations:** ^1^Physical Education Department, College of Education, Qatar University, Doha, Qatar; ^2^Sport Science Program, College of Arts and Sciences, Qatar University, Doha, Qatar

**Keywords:** wearable telemetry body metrics shirt, validity, monitoring heart rate, Polar Team Pro, field test

## Abstract

The aim of the study was to assess the validity and reliability of wearable body metric Hexoskin “smart shirt” in measuring heart rate (HR) at pre-exercise and during peak effort in a field test incorporating vigorous movements of the upper body. Measurements were recorded simultaneously using the Hexoskin and Polar Team Pro. Nine male professional handball players (age: 21.8 ± 2.4 years; weight: 83 ± 10.26 kg; height: 1.81 ± 0.09 m; and BMI: 25.17 ± 2.23) volitionally participated in the study by completing two 400 m shuttle run test trials (10 shuttles), each separated by a 48 to 72 h recovery period. Results indicated significant correlations between Hexoskin and Polar Team Pro system in pre-exercise HR. Hexoskin provided erroneous measurements in four of the nine athletes during peak effort. Subsequent correction yielded no consistency between the Polar Team Pro system and Hexoskin between the first and the second trial. Hexoskin showed significant reliability in pre-exercise HR. However, Hexoskin picked up excessive artifact during vigorous physical activity in four of the nine athletes rendering the results in these cases useless. Nevertheless, in athletes where artifact was not an issue, ICC yielded a good estimate. The main findings indicate that Hexoskin has good validity and reliability in measuring pre-exercise HR in handball players and hence may be used with high confidence in slow motion activities. However, vigorous physical activity with jarring multidirectional upper body movements posed a challenge for Hexoskin.

## Introduction

Robust, small, and non-obtrusive accurate measurement tracking devices have become increasingly popular in professional sports, recreational exercise, and research (Paradiso et al., 2005). Devices that accurately and reliably monitor physiological, metabolic and technical variables during physical activity without being intrusive and without influencing the mechanics of the athletes can be of great benefit by providing the end-user, trainer, clinician, or coach with useful real-time information in real playing situations. However, the plethora of device choices and inflated manufacturer promises makes it hard to select the right measuring and assessment device for the multitude of physical activities, body positions, athlete morphometry, and environmental conditions. Choosing the ideal technology depends on many factors such cost, mobility, size, and sport specificity. Hence, several observations lend credence to the argument that equipment validity and reliability must be established for each physical activity or sport (Laporte et al., 1985).

The commercially sold Hexoskin shirt (Carré Technologies Inc., San Francisco, CA, United States) is one of the most lightweight and cost-effective (Notley et al., 2018; D’souza et al., 2019) physiological telemetry devices. Hexoskin claims to provide accurate physiological and kinetic data such as heart rate (HR), heart rate variability (HRV), breathing rate (BR), and breathing volume (BV) in real-time via wireless telemetry. The device also measures parameters related to physical activity such as exercise intensity, step count, cadence, and caloric expenditure by utilizing cardiac and breathing sensors imbedded in the shirt. The Hexoskin shirt contains cardiac sensors in their built-in thoracic and abdominal bands.

Although HR is typically the most frequently monitored variable, as it is correlated very highly with training intensity (Djaoui et al., 2017) and is easily measurable, Hexoskin can be useful in measuring the BR and electrocardiogram (ECG) signals during exercise outside of a laboratory setting, which in the past were found challenging to monitor in ecological conditions. This is problem mostly manifest in vigorous upper body movements, where the sensors are located. These challenges limit the use of such monitors to evaluate medical conditions or monitor untoward responses to vigorous or high intensity physical exercise (Fieselmann et al., 1993; Yuan et al., 2013).

Villar et al. (2015) studied the validity of the Hexoskin by comparing it to “gold-standard” devices and found that it provided valid and consistent results for walking, standing, sitting and laying supine with high intraclass correlation (ICC) for HR, BV, and hip motion intensity. Moreover, Elliot et al. (2019) concluded that Hexoskin was adequately valid and reliable for the measurement of HR during maximal aerobic power testing with elite cyclists but advised that calculated minute ventilation measured during cycling should be interpreted with caution due to lower validity and reliability. Other studies supported the validity and reliability of the Hexoskin wearable body metrics telemetry shirt in measuring HR during moderate and vigorous intensities (Montes et al., 2015, 2018; Al Sayed et al., 2017; Phillips et al., 2017; Smith et al., 2019).

The ecological validity and reliability of this device warrants further scientific investigation, particularly during maximal exercises with unstudied body movements and in various athletic populations. Therefore, the purpose of the present study was to examine the validity and reliability of Hexoskin in measuring pre-exercise and peak HR by comparing it to Polar Team Pro in professional handball players during game-like settings.

## Materials and Methods

### Participants

Nine senior male professional handball players among the same team from Qatar Handball League (age: 21.8 ± 2.4 years; weight: 83 ± 10.26 kg; height: 1.81 ± 0.09 m; and BMI: 25.17 ± 2.23) voluntarily participated in the research. The study was conducted according to the Declaration of Helsinki and the protocol was fully approved by Qatar University-IRB before subject recruitment and data collection (QU-IRB190-A17). Prior to the study, all participants read and signed a written informed consent form that detailed potential risks and benefits, listed the methods and procedures, and addressed data confidentiality associated with this study. Participants were informed that they could withdraw from the study at any time without penalty. A medical and training history questionnaire (age, height, body mass, training characteristics, history of injuries, and playing experience) was first completed. Consequently, the team physician performed a physical and orthopedic examination focusing on medical conditions that might preclude players from participating in maximal testing. All athletes were cleared for participation in the study.

The study was conducted over a period of 10 weeks (from January to March). By virtue of the nature of their sport and time-in-season, participants were accustomed to moderate strength training (1 session per week of bench press and half squat exercises at 60–80% of one-trial maximum) and had participated in the standard training program from the beginning of the competitive season in September. This routine consisted of six 90-min training sessions per week, plus a competitive game played at the weekend. Physical conditioning, conducted three times per week, was aimed at strength and power development and incorporated high-intensity interval training, weight-lifting, plyometrics, power lifting, and general calisthenics. Anaerobic training consisted of plyometric and sprint training drills, and aerobic fitness was developed using small-sided games. Training sessions consisted mainly of technical-tactical skill development (60% of the session time) and strength and conditioning routines (40% of the session time).

All tests were be administrated by the same ACSM-certified examiner. Dietary intake was maintained as homogeneous as possible for all players during the testing days. Before assessment, players were asked to refrain from consuming stimulants (e.g., caffeine) or depressants (e.g., alcohol) substances. To avoid dehydration, *ad libitum* hydration was allowed during all testing sessions.

### Procedures

Players were fitted with the Polar Team Pro-chest belt and Hexoskin shirt. A 2 cm gap was kept between the cardiac sensor of the Hexoskin and the Polar Team Pro sensor to avoid electrical or mechanical interference. To stabilize the Hexoskin shirt, two elastic straps were fastened around the chest. A dab of glycerin-based cream was used on the cardiac textile electrodes as recommend by Hexoskin manufacturer. Both devices were synchronized to their accompanying applications to capture live data on iPads. HR was extracted from the ECG and recoded in units of beats per minute (bpm).

The participants were familiarized with the procedures and evaluation methods. During training, players were initially fitted for the Hexoskin shirt based on the sizing chart provided by the manufacturer. They were then asked to lay supine for 5 min to measure pre-exercise HR. After performing a standardized warm-up, players completed a 400 m shuttle run test (10 shuttles). The shuttle trial was repeated 48 to 72 h later. All assessments were be conducted at the same time of the day (6 p.m.) to control the effects of diurnal variation on performance.

### Statistical Analysis

Statistical analyses were performed using SPSS version 25.0 for Windows (SPSS Inc., Chicago, IL, United States). Measures of central tendency and measures of variance were calculated for all variables. Shapiro–Wilk test and Levene’s test were used to verify the normal distribution and homogeneity of the parameters, respectively. Pearson product moment correlation coefficient (validity coefficient) was used to determine the convergent validity between Polar Team Pro and Hexoskin results. Meaningfulness of correlations was evaluated using the Hopkins’ (2000) classification: *r* < 0.1, trivial; 0.1–0.3, small; 0.3–0.5, moderate; 0.5–0.7, large; 0.7–0.9, very large; >0.9, nearly perfect; and 1 perfect.

Bland and Altman plots were derived to determine the agreement between the two tools (i.e., Hexoskin short and Polar Team Pro) to measure pre-exercise HR and peak HR. In these plots the mean difference (d) between the measurements from Hexoskin and Polar Team Pro with the 95% limits of agreement (LOA) were presented (Bland and Altman, 1986, 2010). Regression linear analyses (Dependent variable: difference between measurements; Independent variable: mean of the measurements) were then performed to determine whether any proportional bias occurred. These two methods (Pearson product moment correlation coefficient and Bland and Altman plots) give complementary information as highlighted by Atkinson and Nevill (1997) and Lawrence and Chinchilli (1997).

Relative reliability was assessed using the intraclass correlation coefficient (ICC) between test and retest values at a similar metabolic effort. The ICC indicated an excellent relative reliability if the value was above 0.75, fair-to-good reliability between 0.40 and 0.75, and poor reliability when less than 0.40 (Portney, 2020). ICC values can be affected by inter-subject variability of scores, because a large ICC may be reported despite poor trial-to-trial consistency if the interrater variability is too high (Shrout and Fleiss, 1979; Hopkins, 2000). *p* value will be used to evaluate the accuracy and significance of the ICC. The standard error of measurement (SEM) and the Bland and Altman analysis were used to analyze the agreement and absolute reliability at a similar metabolic effort. In a Bland Altman plot, the mean of each individual data pair is shown on the *x*-axis and the difference between each data pair on the *y*-axis with the 95% LOA were presented (Bland and Altman, 1986, 2010). Regression linear analyses (Dependent variable: difference between trials; Independent variable: mean of trials) were then performance to determine whether any proportional bias occurred. The *p* value for statistical significance was set *a-priori* at ≤ 0.05.

## Results

### Convergent Validity

Heart rate was initially analyzed using data from all participants (*n* = 9). “Large” to “very large” significant correlations have been shown in pre-exercise HR measured in the first and second trial, respectively, between Hexoskin and Polar Team Pro system results. There were no significant correlations in peak HR measured in both trials (Table 1).

**TABLE 1:** Convergent validity between Hexoskin and Polar Team Pro system results.

	*r*	Meaningfulness	*p*
**Peak HR**			
Trial 1	0.13	Small	0.72
Trial 1 (after removing the abnormal measurements)	0.94*	Nearly perfect	0.01*
Trial 2	0.64	Large	0.06
**HR rest**			
Trial 1	0.76*	Very large	0.01*
Trial 2	0.67*	Large	0.04*

*r*, Pearson product moment correlation coefficient; *p*, significance; HR, heart rate; *Correlation is significant at the 0.05 level.

**FIGURE 1:**
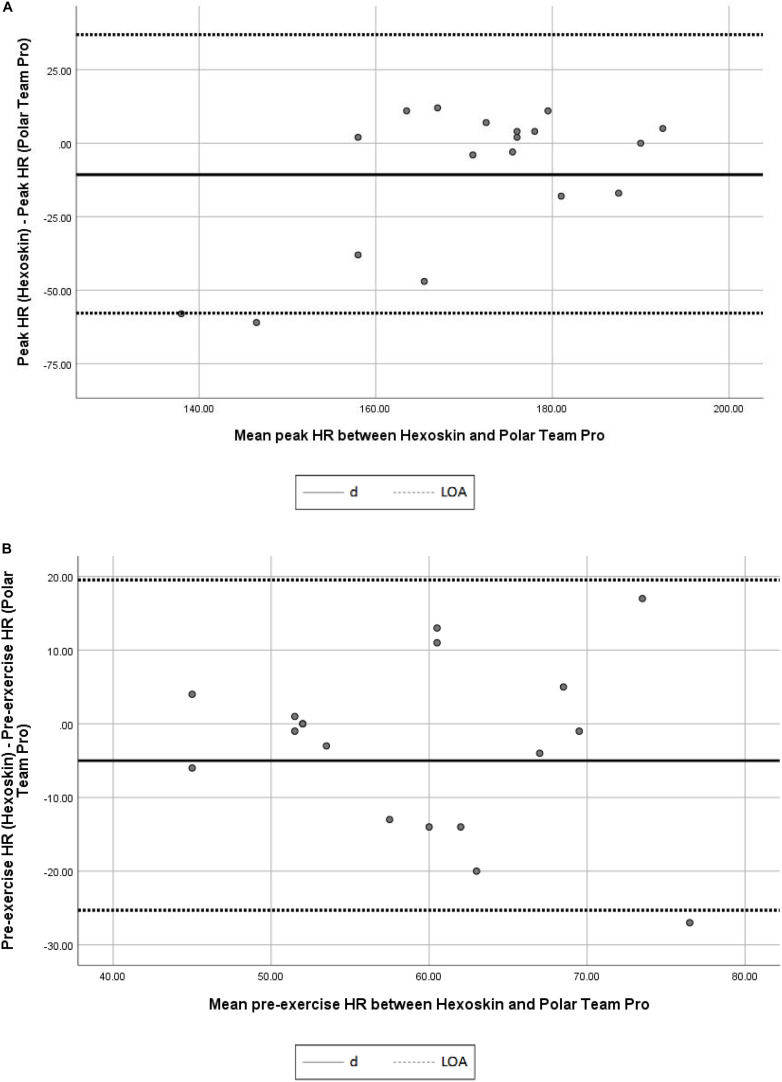
**(A)** Bland and Altman plot concurrent validity of Hexoskin in measuring peak HR. d, mean difference between peak HR (Hexoskin) and peak HR (Polar Team Pro). LOA, limits of agreement; HR, heart rate (batt/min). **(B)** Bland and Altman plot validity of Hexoskin in measuring pre-exercise HR. d, mean difference between pre-exercise HR (Hexoskin) and pre-exercise HR (Polar Team Pro); LOA, limits of agreement; HR, heart rate (batt/min).

**FIGURE 2:**
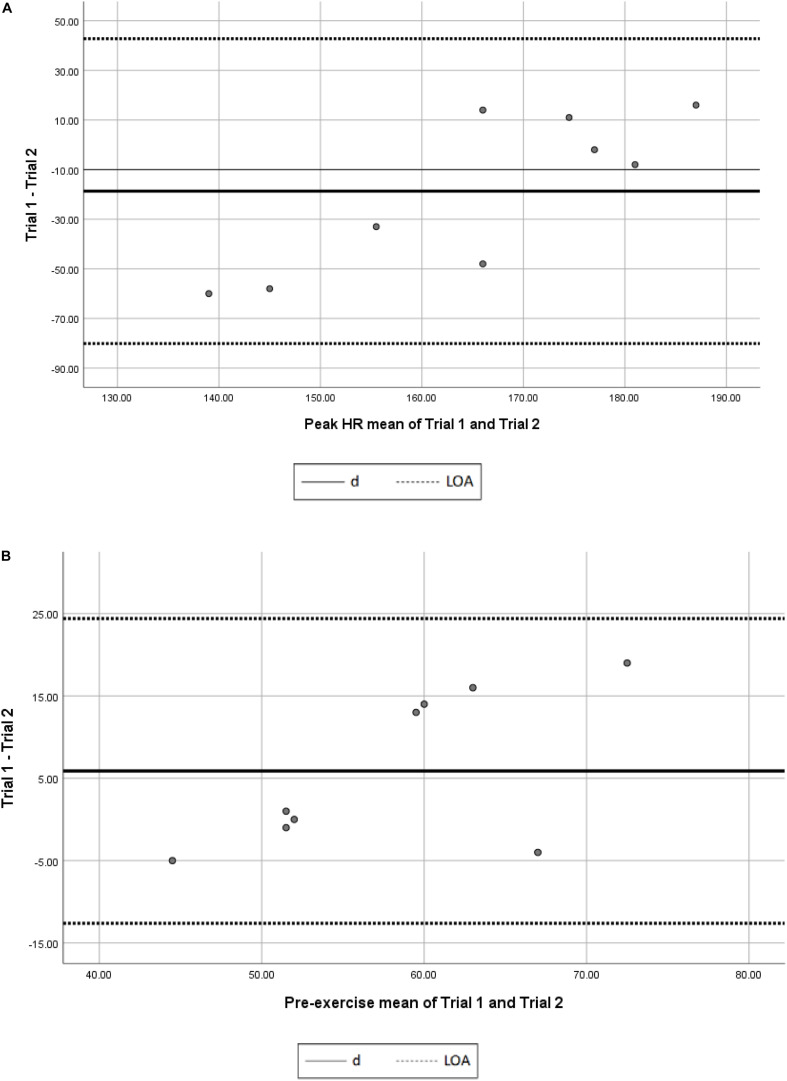
**(A)** Bland-Altman plot reliability of Hexoskin in measuring peak HR. d, mean difference between peak HR trial 1 and 2; LOA, limits of agreement; HR, heart rate (batt/min). **(B)** Bland-Altman plot reliability of Hexoskin in measuring pre-exercise HR. d, mean difference between pre-exercise HR trial 1 and 2; LOA, limits of agreement; HR, heart rate (batt/min).

Figures 1A, 2A present the Bland and Altman plot to determine the agreement between Hexoskin and Polar team pro in measuring pre-exercise HR and peak HR. The linear regression analysis showed a significant relation (*p* = 0.004) between the difference and the mean between Hexoskin and polar team pro in measuring the peak HR indicating the present of a proportional of bias. However, non-significant correlation (*p* > 0.05) has been revealed between the difference and the mean between Hexoskin and polar team pro in measuring pre-exercise HR showing the no proportional bias.

Analyses were then re-conducted after excluding the data from four cases with inaccurate recordings (Table 1). Once the exclusions were made, the correction between the Polar Team Pro and Hexoskin systems increased to “nearly perfect” in the first trial (*r* = 0.94; *p* = 0.01). None of abnormal measurements appeared in the second trial. The correlation between the Polar Team Pro and Hexoskin systems in the second trial is considered “large” and trended toward statistical significance (*r* = 0.64, *p* = 0.06).

### Reliability

Significant “fair-to-good” reliability has been shown in pre-exercise HR measured by Hexoskin (Table 2). However, “poor” and non-significant reliability has been shown in peak HR measured by Hexoskin when considering all results (Table 2). However, after removing the four cases with inaccurate Hexoskin measurements, the reliability was significant and considered as “excellent”. Polar Team Pro system showed “excellent” reliability (above 0.75).

**TABLE 2:** Reliability of Hexoskin and Polar Team Pro system.

	HR ± SD (Trial 1)	HR ± SD (Trial 2)	ICC	*p*	Meaningfulness	SEM
**Hexoskin**						
Peak HR	109 ± 30.65	173 ± 8.64	0.08	0.45	Poor	30.06
Peak HR (After removing the abnormal measurements)	159 ± 9.27	173 ± 8.64	0.92*	0.01*	Excellent	1.07
HR rest	42 ± 12.45	47 ± 6.76	0.71*	0.04*	Fair-to-good	5.08
**Polar team pro**						
Peak HR	161 ± 9.50	157 ± 13.65	0.84*	0.01*	Excellent	3.46
HR rest	48 ± 7.38	43 ± 14.05	0.75*	0.03*	Excellent	4.98

ICC, intraclass correlation coefficient; *p*, significance; SEM, standard error of measurement; SD, standard deviation; HR, heart rate; *Significance at the 0.05 level.

Figures 1B, 2B present the Bland and Altman plot to demonstrate the agreement between the first and second trial of measuring pre-exercise and peak HR using Hexoskin. The linear regression analysis displayed a non-significant relation (*p* > 0.05) between the difference and the mean of the first and second trials of pre-exercise HR measurements indicating the absence of a proportional of bias. However, significant correlation (*p* = 0.005) has been revealed between the difference and the mean of trials of peak HR measurements showing the existence of proportional bias.

## Discussion

The aim of the present study was to assess the ability of the wearable body metric Hexoskin shirt to measure the pre-exercise and peak HR in a maximal field test incorporating vigorous movements of the upper body among professional handball players.

The results of the correlations between Polar Team Pro and Hexoskin, using Person’s coefficient, showed that the two systems were significantly correlated when measuring pre-exercise HR (*r* = 0.67–0.76; Large to very large significant correlations; *p* ≤ 0.05). Bland and Altman plot and the regression linear analysis showed the absence of proportional bias. Those results of pre-exercise HR are consistent with prior validation studies of the Hexoskin (Montes et al., 2015, 2018; Al Sayed et al., 2017; Phillips et al., 2017; Smith et al., 2019). Al Sayed et al. (2017) found a high similarity between Hexoskin and Polar systems in measuring HR under different climate conditions and levels of physical exertion (Degrees of the correlation > 0.70). In an another recent study (Smith et al., 2019) Hexoskin showed low discrepancies in measuring HR at all levels (rest, submaximal, and maximal exercise levels) when compared to the “gold stand” laboratory equipment. The discrepancies were less than 10%, which is deemed acceptable for submaximal field use.

However, peak HR demonstrated non-significant correlations (*p* > 0.05) between Hexoskin and Polar Team Pro system results in present study (all cases) contrary to previous studies validating the Hexoskin while riding a bicycle (Al Sayed et al., 2017; Smith et al., 2019) and running on a treadmill (Cherif et al., 2018) to reach peak exercise. Furthermore, Bland and Altman plot and the regression linear analysis showed the existence of proportional bias. The difference might be due the type of body motion used to reach the peak HR. In the study of Al Sayed et al. (2017) and Smith et al. (2019) the test used was cycling on stationary bikes from sitting to high intensity workouts which does not take into consideration the movements of the upper body. In the present study, a 400 m shuttle run test (10 shuttles) was used, where vigorous thoracic twisting movements of the upper body may have caused temporary but frequent sensor-to-skin detachment of Hexoskin and leading to erroneous measurements. It is the same hypothesis of Cherif et al. (2018) who pointed out that that bouncing may have affected the cardiac sensor and resulted in an abnormal recording. Under controlled conditions such as laboratory-based research, permanent control of straps might be possible. However, this is not a realistic ecological solution for monitoring exercises in the field, as in the present study.

Montes et al. (2018) mentioned that some of erroneous HR measurements using Hexoskin might be due to the fit of the shirt. Even during walking, Montes et al. (2018) observed that participants who appeared to have smaller or larger than average chest girths had more issues maintaining consistent HR measurements. Montes et al. (2018) suggested that smaller chests, particularly flat chests, might allow Hexoskin ECG sensors to shift on the skin and bunch up even with the self-hooking elastic strap holding them down. The larger chest girth appeared to either gradually shift them to a position on the skin where they had trouble detecting the HR or created a gap between ECG sensor and the skin, even with self-hooking elastic strap pushing then down. Since professional handball players tend to have large chest girth measurements (Mesosternale; Massuça and Fragoso, 2013; Massuça et al., 2014), the anthropometric attributes of this athletic population might offer another explanation of the invalid measurements of HR using Hexoskin. Smith et al. (2019) highlighted that the failure to properly record HR throughout the cycling exercise may be related to improperly fitting shirts causing the sensors to detach from the skin. Additionally, Elliot et al. (2019) offered the same rationalization in their study where cyclist torso movement during testing increased the likelihood of sensor detachment and offered a caveat when using the Hexoskin at higher intensity due to the lower validity. All the above cited research suggests that sensor-to-skin improvements as well as software programming are needed to ensure proper contact during vigorous movements of the upper body, or bouncing movements where skin displacement may create unfiltered artifact that yields erroneous readings.

Bland and Altman plots to determine the agreement between the first and second trial of measuring HR using Hexoskin showed the absence of proportional bias in pre-exercise measurements. However, it shows the existence of proportional bias in peak HR measurements. Interestingly, after removing those abnormal measurements, results have been changed significantly to be “nearly perfect” (*r* = 0.94; *p* = 0.01) between Polar Team Pro and Hexoskin systems. Furthermore, the good ICC of Hexoskin in measuring the pre-exercise HR confirmed that Hexoskin presents a valid and consistent tool to tracking the HR in the supine resting position. After removing the abnormal measurements, the ICC remains significant and considered as “excellent” (ICC > 0.75). SEM were very low in measuring both HR rest and peak HR. Those results are consistent with the study of Smith et al. (2019) who showed an overall low variability of Hexoskin shirt. That shows the relative and absolute reliability of Hexoskin.

## Conclusion

Hexoskin showed significant reliability and validity in measuring pre-exercise HR compared to Polar Team Pro in professional team handball players. However, in a large proportion of the subjects we tested the validity and reliability of the device was highly suspect. In the present investigation, Hexoskin provided erroneous results in 44.5% of the cases studied. We speculate that technical limitations and artifact management were major contributing factors. In conclusion, Hexoskin may be used with confidence for resting, low impact, and low intensity physical activities. However, more improvements need to be made to improve the validity and reliability of the device during vigorous or jarring open-chain or closed chain exercises, especially those that involve jarring or twisting movements of the upper body.

## Data Availability Statement

The original contributions presented in the study are included in the article/supplementary material, further inquiries can be directed to the corresponding author.

## Ethics Statement

The studies involving human participants were reviewed and approved by Qatar University-Institutional Review board. The patients/participants provided their written informed consent to participate in this study.

## Author Contributions

MH and ZA contributed to the conceptualization, project administration, and visualization. MH and KB contributed to the formal analysis. MH, FD, MK, AM, and ZA contributed to the funding acquisition, investigation, and methodology. MH contributed to the writing of the original draft. MH, SH, ZA, and KB contributed to the review and editing of the writing. All authors contributed to the article and approved the submitted version.

## Conflict of Interest

The authors declare that the research was conducted in the absence of any commercial or financial relationships that could be construed as a potential conflict of interest.
